# Sensitivity and specificity of a low-cost screening protocol for identifying children at risk for language disorders

**DOI:** 10.6061/clinics/2020/e1426

**Published:** 2020-04-06

**Authors:** Daniela Cardilli Dias, Silmara Rondon-Melo, Daniela Regina Molini-Avejonas

**Affiliations:** I Departamento de Fisioterapia, Fonoaudiologia e Terapia Ocupacional, Faculdade de Medicina (FMUSP), Universidade de Sao Paulo, Sao Paulo, SP, BR

**Keywords:** Primary Health Care, Child Language, Language Disorders, Risk Factors, Sensitivity and Specificity

## Abstract

**OBJECTIVES:**

To compare the diagnostic accuracy of a low-cost screening test for identifying children at risk for language disorders with that of a specific language assessment.

**METHODS:**

The study was conducted during a polio vaccination campaign in basic health units in western São Paulo, Brazil. The parents/guardians of 1000 children aged between 0 and 5 years were asked to answer questions of a specific screening test. The instrument consisted of a uniform set of questions about the main milestones in language development (from 0 to 5 years of age) with scaled scores to assess responses. There were no exclusion criteria. After the screening test, the children were referred to a specific language assessment by ABFW, following a determined flow of referrals. The results obtained in the screening were compared to those obtained in the specific language assessment; then, the sensitivity, specificity, accuracy, and positive and negative predictive values were determined for the screening test. Children who failed the screening test also underwent an audiological evaluation. The statistical significance was set at 5%.

**RESULTS:**

The majority of the participants were aged between 4 and 5 years (21.82%) and were male (51.6%). The sensitivity and specificity values were 82.5% and 98.93%, respectively. The area under the curve was 0.907 (0.887–0.925), and the screening test showed 96% accuracy.

**CONCLUSIONS:**

The screening test showed high diagnostic efficiency in determining the risk of language disorders in children aged between 0 and 5 years.

## INTRODUCTION

Knowledge on the prevalence, incidence, and risk factors of a particular disease in the community is important for health professionals and managers to allocate sufficient resources for managing the problems associated with that disease and to design and implement health promotion and disease prevention strategies 1. In Speech-Language and Hearing Sciences (SLHS), it is known that epidemiological studies can be used for correct and early identification of the risk of communication disorders in a given population. The results of such studies allow better programing of health actions related to disease prevention and rehabilitation of the population identified to be at risk of or having communication disorders 1-3.

Regarding epidemiological studies, the SLHS literature shows that speech-language delay and language disorders are the most common developmental difficulties in childhood 1,4-11. According to the American Speech-Language and Hearing Association (ASHA) 11, 10% of children have some form of language impairment, while the prevalence rates of phonological disorders and specific language impairment among preschool children is between 2 and 19% and 7%, respectively.

Considering that between 40% and 60% of language disorders go untreated and the negative effects of language impairment on an individual's social development and education 7,12-14, the use of specific, standardized, and validated instruments for early detection of the risk of language delay or impairment is important. However, validation studies on the sensitivity and specificity of methods of early detection of the risk of language delay are scarce 1,7-9,15,16.

Konning et al. 17 conducted a cluster-randomized trial to assess the screening performance of a specific language-screening instrument in a large sample of children aged between 18 and 24 months in the Netherlands. The results indicated that 55% of screening-positive children had confirmed language delay and the estimated sensitivity of the screening test ranged between 24% and 52%, depending on the severity of the language disorder.

A study conducted in Brazil investigated the risk factors of language disorders in children aged between 0 and 5 years through a specific survey and conducted follow-up with a screening test for communication disorders 15. The authors found that the average length of time between when parents first suspected their child to have a communication disorder and the first speech-language screening was 16 months; the results also indicated that the average age of detection of risk factors was 4 years. These findings reflect the difficulties involved in early detection and intervention for preventing communication disorders.

A recent literature review that investigated the predictive validity of preschool screening tools for language and/or behavioral difficulties indicated that the language development surveys administered at 2 years of age achieved the best predictive validity, with 67% sensitivity, 94% specificity, 88% negative predictive value, and 80% positive predictive value 18. The authors also found that language-screening tools were more effective and achieved higher sensitivity and positive predictive value than either behavior or combined screening tools (language and behavior) for identifying children with language difficulties.

There are some limitations to the studies that proposed population surveys as screening instruments, such as a generalized and superficial view of language development, low levels of sensitivity, specific focus on understanding and expressive language skills, and the use of sample populations with a small number of participants and no diversity in age groups 9,19. Another limitation is that although the screening tools were widely used for language development 7,20,21, the majority were poorly validated 18.

Recent studies suggest that there is insufficient scientific evidence on the effectiveness of screening tests and speech-language assessments for children aged up to 5 years without suspected disorders 7,22. These studies underline the need for further research to determine the reliability, sensitivity, and specificity of specific low-cost instruments before promoting their widespread adoption 1,6,7,23.

It is important to consider the evidence that indicates the benefit of including a specific language-screening instrument in routine screening performed at child health centers; this can lead to early detection of language delay, enabling informed referrals to specialist services and early intervention 17,18.

Therefore, the objective of this study was to compare the diagnostic accuracy of a low-cost screening test for identifying children at risk for language disorders with that of a specific gold standard language assessment.

## MATERIALS AND METHODS

This study was approved by the research ethics committee of the School of Medicine at the University of São Paulo (FMUSP, acronym in Portuguese) (CAPPesq HCFMUSP 387/10).

The study was conducted during a polio vaccination campaign in basic health units in western São Paulo, Brazil. The parents/guardians of 1,000 children aged between 0 and 5 years were asked to answer the questions of a specific screening test. The children's parents/guardians who agreed to participate were asked to sign an informed consent form. There were no exclusion criteria.

The study was conducted with 1,000 children aged between 0 and 5 years living in the micro-region Butantã/Jaguaré in the municipality of São Paulo, Brazil, and treated in health centers located in western São Paulo during a vaccination campaign.

The instrument used in the present study was a screening test for language disorders developed and validated by the ASHA 24. The screening contains specific questions about language development (language production and understanding) considering the main milestones according to age – from zero to five years old (Appendix).

After obtaining the ASHA's formal license, the process of translation and back-translation of the protocol was initiated, according to the requirement of standardization of the test and guidelines for the process of cross-cultural adaptation (translation, synthesis, back-translation, expert committee review, and pretesting) 25. The translation and back-translation were carried out by a Brazilian individual fluent in English and reviewed by two other native Brazilian individuals fluent in English, both speech-language pathologists and public university professors. These professionals not only translated but also socioculturally adapted the test for expressions, names, and adequate examples in Brazilian Portuguese.

To check the reliability of the translated questions and their format, a bilingual speech-language pathologist (fluent both in Portuguese and English) performed the back-translation of the questionnaire (English to Portuguese). Both versions were compared to ensure their equivalence. In the back-translated version, the translator carried out further adaptations for vocabulary, syntactic issues, and expressions and then compared it to the published English version. The shortcomings and errors were corrected, and the first draft was prepared.

The first draft was sent to a committee (four speech pathologists experts including two professors, one specialist, and a grammarian and linguist) that thoroughly evaluated the first version of the screening test for simplicity of the text, grammar, and use of proper terms and syntax. Four steps of equivalence, namely semantic, idiomatic, experiential, and conceptual equivalence, were evaluated for cross-cultural adaptation 25.

Finally, the judges examined the committee's considerations, accepting pertinent suggestions and elaborating the final version to be used in the pre-test. The pre-test involved ten participants with different levels of education, from complete primary school to graduate degree. The participants were asked to read, paraphrase, and comment on their understanding of the instructions, items, and response options of the instrument. Based on their responses, no further modification was needed.

We used face-to-face interviews for data collection to avoid selection bias related to illiterate participants and to reduce the number of non-respondents. All participants were interviewed by a trained interviewer individually. To assess test-retest reliability, 30 patients completed the screening over 2 weeks; the procedure was conducted by the same interviewer.

Cronbach's alpha and intraclass correlation coefficient (ICC) were used to determine the reliability of the screening; the Cronbach's alpha coefficient was ≤0.7 and the ICC was ≥0.85.

The screening process examined the main developmental milestones across the language domain using age-specific questions. The participants were divided into the following age groups according to the ASHA criteria 24:

- Group I: 0 to 3 months
- Group II: 4 to 6 months
- Group III: 7 months to 1 year
- Group IV: 1 year and 1 month to 2 years
- Group V: 2 years and 1 month to 3 years
- Group VI: 3 years and 1 month to 4 years
- Group VII: 4 years and 1 month to 5 years and 11 months.

The answer options for each screening question were “no,” “yes,” or “no answer” (when the child parent/guardian was unsure about the answer). Each answer was scored as per the ASHA criteria in the original version of the screening protocol (Table 1).

Each interview lasted between 5 and 15 minutes. At the end of the screening process, each participant was informed of his/her final score, which was the sum of the scores obtained for each question.

All children who underwent the screening test were referred to a specific speech-language assessment in the school-clinic of the undergraduate speech therapy program of the School of Medicine at the University of São Paulo (FMUSP).

The speech-language assessment was performed using the ABFW test 26. The ABFW test allows the tester to obtain quantitative data and compare them with reference values for typical speech and language development in Brazilian Portuguese-speaking children, considering four specific subareas: phonology, vocabulary, fluency, and pragmatics. The phonology area is composed of a qualitative and quantitative assessment of the child's phonetic inventory and the classification of phonological processes, when identified (14 types). The vocabulary area consists of quantitative and qualitative assessment of nine conceptual fields related to expressive vocabulary: food, transport, furniture and utensils, professions, places, shapes and colors, toys, and musical instruments. The fluency area is composed of qualitative and quantitative evaluation of three areas: typology of speech disruption, speech velocity, and frequency of speech disruption. Finally, the area of pragmatics is composed of qualitative and quantitative assessment of functional communication skills in three areas: number of communicative acts, means, and functions. It is a test that aims to draw an overall profile of the areas it covers and support the diagnosis of speech and language disorders. The average time required for the ABFW test is 90 minutes, and the time for complete data analysis is usually 6 hours.

A standardized test validated for use in Brazilian Portuguese-speaking children does not exist, and the ABFW test 26 is currently the most reliable test since it provides a reference range that serves as a basis for comparison with “normality.”

The data were recorded using individual protocols and on video and analyzed by two speech-language pathologists (SLPs) specializing in speech-language assessment. They received specific training in analyzing the results of the ABFW test. The SLPs were blinded to the study objectives. Data analysis was completed in 2 months after screening and within a maximum of 2 weeks after the language assessment. Children who presented altered parameters for at least one of the four areas covered by the ABFW test were considered as to present with language development disorders.

Children who failed the screening test, independently of the results in the language assessment, also underwent a comprehensive audiological evaluation using audiometry and immitanciometry. Children who had a hearing threshold of up to 15 dB HL for all tested frequencies (250 to 8000 Hz), type A tympanogram curve, and showed acoustic reflex were considered to have normal hearing 6. Children who failed in at least one of the two audiological tests were referred to an otolaryngological assessment. The parent/guardian was responsible for accepting the referral.

Data on the age and sex composition of the sample were analyzed using Student's *t*-test, at a significance level of 5%.

The specificity and sensitivity of the instrument were determined based on the relationship between the answers obtained by the screening instrument and the results of the specific speech-language assessment (gold standard criteria).

In accordance with the definitions proposed by Agresti 27, sensitivity was defined as the likelihood of a given participant “failing” the screening test, where the child exhibited “delayed development” based on both the ASHA instrument criteria (at least 50% negative answers in the language production and understanding dimensions) and results of the comprehensive speech-language assessment; specificity was defined as the likelihood of a given participant “passing” the screening test, where the child was considered to exhibit “normal” development based on both the instrument criteria (at least 50% positive answers in the language production and understanding dimensions) and results of comprehensive speech-language assessment.

The positive predictive value (PPV) was based on the ratio between the number of true positives (TP) and the sum of TP and false positives (FP), where PPV=TP/TP+FP. The negative predictive value (NPV) was calculated based on the ratio between the number of true negatives (TN) and the sum of TP and false negatives (FN), where NPV=TN/TN+FN.

Accuracy (A) was calculated based on the ratio between the sum of VP and VN and the total number of cases analyzed in the study (N), where A=TP+TN/N. The area under the receiver operating characteristic (ROC) curve was also calculated.

The likelihood ratio for positive test results (PLR) was calculated based on the ratio between sensitivity (S) and one minus specificity (E), where PLR=S/1-E, while the likelihood ratio for negative test results (NLR) was calculated based on the ratio between one minus sensitivity and specificity, where NLR=1-S/E.

For the analysis of sociodemographic factors of children who failed the language assessment (gold standard, N=120), the criteria established by the *Associação Brasileira de Empresas de Pesquisa* 28 were used. These criteria enable the classification of the families into economic classes considering the existence/absence and number of household items, such as television, radio, bathroom, automobile, domestic servant, washing machine, VCR or DVD player, refrigerator, and freezer, and family income. The corresponding family income established for each economic class was as follows: A1 (US$4991.00), A2 (US$3606.00), B1 (US$2066.00), B2 (US$1154.00), C1 (US$639.00), C2 (US$418.00), D (US$295.00) and E (US$180.00).

## RESULTS

The study included 1000 children: 516 boys (51.6%) and 484 girls (48.82%). Table 2 shows the composition of the sample according to age and sex.

The 4–5-year age group had a larger number of participants than the other groups. This difference was statistically significant (n=218; *p*<0.009).

The reliability of the screening tool is explained in Table 3, which indicated optimal agreement between the test and retest.

**Figure 1:**
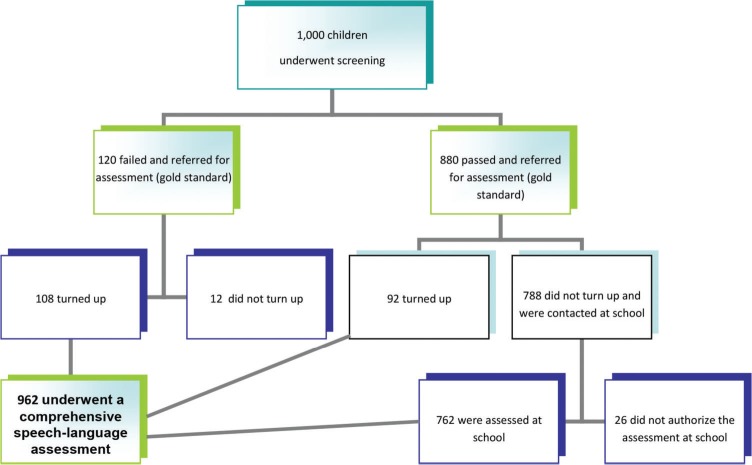
Results of the screening process and referrals.

The results of the screening process and referrals are presented in Figure 1. It is important to highlight that all the children were referred to a specific language assessment at a SLHS Clinic at a major university, but the majority of the children who passed the screening did not turn up (probably because the parents had no complaints). Therefore, the speech-language pathologist went to the childreńs schools to assess them. The time-interval between the screening and assessment was 1 month. These results are presented in Figure 1.

The relationship between the children who failed or passed the screening and those who failed or passed the language assessment (contingency) is presented in Table 4.

**Figure 2:**
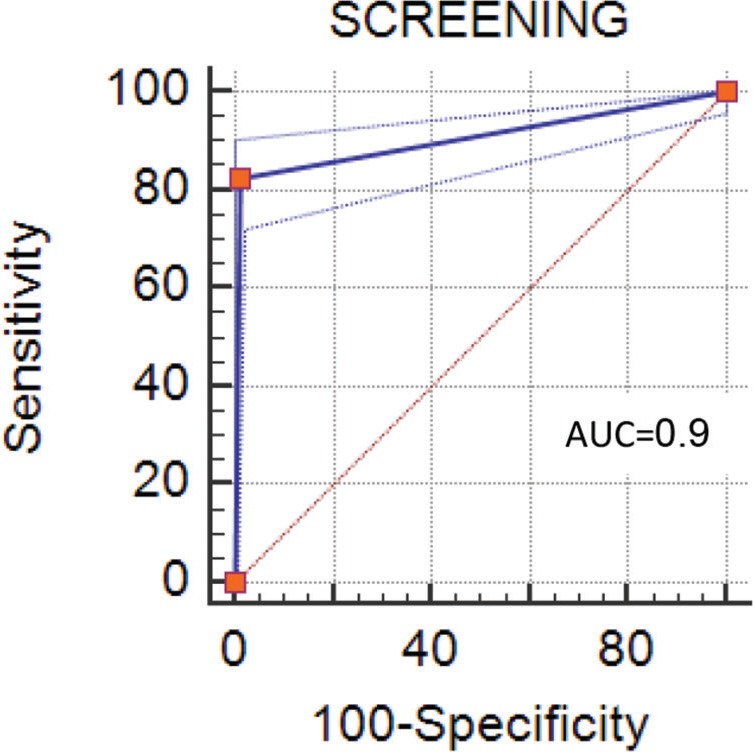
ROC curve.

The sensitivity and specificity values, predictive value, and AUC (0.907 [0.887–0.925]) value may be considered high and satisfactory 29-31 (Figure 2).

The PLR and NLR were 77 and 0.18, respectively. The fact that the PLR value was greater than 1 and the NLR value was very close to 0 indicates a high likelihood of the instrument identifying true positives and negatives (Table 5).

**Figure 3:**
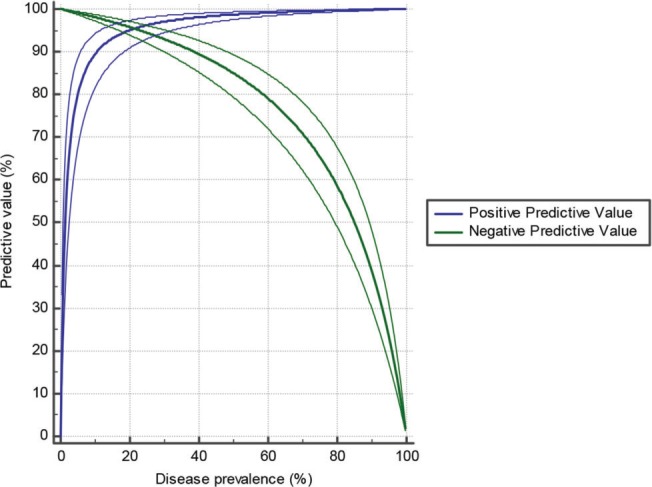
Plot *versus* prevalence.

Using the same screening tool in a population with high disease prevalence increases its positive predictive value. Conversely, increased prevalence results in a decreased NPV. Figure 3 depicts the relationship between disease prevalence and predictive value.

All participants who underwent an audiological evaluation presented a hearing threshold that fell within normal limits (as per criteria described in the methods section).

The sociodemographic characterization of the 120 children who failed the language assessment is presented in Table 6; 69% of the children were male.

## DISCUSSION

The screening instrument tested in this study showed high sensitivity (82.5%) and specificity (98,93%) and high AUC (0.90) for detecting the risk of language delay in children aged between 0 and 5 years. An international validation study on a widely used language-screening protocol for children aged between 18 and 24 months showed that the test sensitivity ranged between 24% and 52% (acceptable) and that the specificity ranged between 97% and 98% (high) 17. Another study comparing five different screening instruments for language delay showed that the sensitivity of the tests ranged between 42% and 86% with AUC values between 0.75 and 0.87. The findings also show that shorter tests had higher sensitivity and accuracy 9. Therefore, the short time needed for the screening test evaluated in the present study may have directly influenced the sensitivity and specificity values, demonstrating the accuracy and efficiency of this instrument in detecting the risk of language delay in the population under consideration.

The sample comprised more male children than female children. This difference was statistically significant in the 1–2-years age group. The 2–3-years age group was the only group where there were more girls than boys. This difference was statistically significant. The results also show that more boys failed the test than girls. This difference was statistically significant, corroborating the findings of previous studies where the prevalence of the risk of communication disorders was found to be greater among boys than among girls 4,5,10,15,32.

The 4–5-years age group had a larger number of participants than the other groups. This difference was shown to be statistically significant. International studies have reported that the prevalence of language delay and/or language disorders among children aged between 2 and 5 years ranges between 2.4% and 12% 17,33. Another international study showed that the prevalence of speech and language delay in children aged 8 years was 5.9% 33. National studies have shown that the prevalence of communication disorders is high in the 3–8-years age group and that the most critical age is between 4 and 6 years 4,8,10.

A comparison of the results of the present study with the findings of previous studies suggests that the predominance of participants aged between 4 and 5 years in the sample may reflect difficulties in the early detection of problems in children at risk of or children with language delay in the region in which the study was undertaken. Hence, there is an urgent need to evaluate suitable screening instruments and promote the wider use of validated tests from the early stages of child development to minimize the risks of language delay 17,22.

We found an estimated prevalence rate of language delay/disorder of 11% in the screening and a true prevalence rate of 12.5% by the gold criteria. The median prevalence of receptive language delay/disorder ranged from 2.63% to 3.59%, that of expressive language delay/disorder ranged from 2.81% to 16%, and that of combined receptive and expressive language delay/disorder ranged from 2.02% to 3.01% 32,34. According to the ASHA 11, 10% of children have some form of language disorder. In Brazil, a recent study identified approximately 10% of complaints related to speech-language and hearing disorders in a population living in a low-income region of the city of São Paulo that was treated in primary health care units 35. Another study conducted a survey with parents/guardians of 525 children in primary health care units and identified that oral language disorders had a prevalence of 15% in children 36. The results of a recent British study identified that language disorders had an estimated prevalence of 9.92% in children aged 4–5 years 37.

Of note, the reasons for this variability in the prevalence of speech and language disorders include the definition of what constitutes a case, the severity and type of communication disorder that is included in the definition, the nature of the surveyed population, and differences in methodological procedures 33,38,39.

Studies suggest that questionnaires offer an effective, rapid, and low-cost method for determining the prevalence of a given disorder in large groups 1,40-42. The use of low-cost screening methods like the one proposed in this study can lower the demand for unnecessary procedures, thus reducing health care costs and improving the efficiency and effectiveness of service delivery 6. In Brazil, there is no low-cost protocol for identification of the risk of language disorders similar to the one presented in this study. The current protocol is easy to apply and has high sensitivity and specificity; it can also be applied by professionals who work in primary health care settings, such as community health agents, and not just specialists. All these advantages of the instrument enable its application to large populations 35-40.

The adherence rate among individuals referred for comprehensive assessment was high, particularly among children who failed the screening test, thus contributing to high levels of sensitivity and specificity. One of the factors that may have contributed to the loss of participants in this stage of the study was the distance between the medical center and the patient's home and the resulting cost and traveling time required to undertake the assessment, which has been shown to affect adherence to follow-up health care 6,43. We solved this issue by assessing the children in their schools.

Another limitation of this study is that the speech and language assessment used in this study was not a national or international gold standard protocol. However, a gold standard protocol for detecting speech and language disorders in Brazilian Portuguese speaking children does not exist. The lack of a gold standard protocol for this type of evaluation is a limiting factor in the development of effective screening tools for the detection of language delay in children 7,17. The variable nature of language development in the first years of a child's life and of child development as a whole means that it is difficult to develop a standardized definition of normality and delay, which ultimately results in the establishment of evaluation criteria by consensus 9,17,22. The present study proposes a rapid, low-cost screening test for the detection of language delay in Brazilian Portuguese-speaking children aged between 0 and 5 years, which was shown to have high sensitivity and specificity. Further research is recommended to confirm and generalize the results by testing the screening instrument in other regions of Brazil and adapting it for use with children who speak other languages and with children from different socioeconomic backgrounds, as suggested by previous studies. In addition, it is important to determine the sensitivity and specificity of the screening test for detecting different degrees of speech and language disorders based on the results of speech and language assessment and for early detection of risk and/or delay during the language development process 17,22.

## CONCLUSION

Our findings show that the screening test proposed in this study has high sensitivity (82,5%) and specificity (98,93%) for detecting the risk of language delay in children aged between 0 and 5 years. The true prevalence was 12.47%. The screening test is an easy-to-use, rapid, low-cost method for screening large numbers of children in primary health care settings.

Further research with larger samples consisting of children from different regions of Brazil and involving the adaptation of the instrument for use in children who speak other languages is recommended to determine the level of sensitivity and specificity of the instrument and generalizability of the results.

## AUTHOR CONTRIBUTIONS

Dias DC was responsible for the acquisition and analysis of data, drafting of the manuscript and literature review. Rondon-Melo S provided substantial contribution to the analysis and interpretation of data and final draft of the manuscript. Molini-Avejonas DR was responsible for the manuscript concept and design, acquisition, analysis, and interpretation of data, manuscript drafting, critically review for important intellectual content and approval of the final manuscript version.

## Figures and Tables

**Table 1:** Screening score attribution and pass/fail criteria.

Type of answer	Score	Pass/fail criteria
No	-1	Negative majority: fail
Yes	1	Positive majority: pass
No answer/neuter	0	

**Table 2:** Composition of the sample according to age and sex.

	Sex			
Age	Male %	Female %	Total %	*p*-value
0-3 months	7.13	7.13	14.26	0.905
4-6 months	5.43	4.40	9.84	0.150
7-12 months	4.39	3.44	7.84	0.200
1-2 years	9.84	7.78	17.58	0.030*
2-3 years	5.4	8	13.40	0.001*
3-4 years	7.7	7.55	15.26	0.500
4-5 years	11.29	10.52	21.82	0.560
Total	51.6	48.82	100	
*p*-value	0.420			

* *p*<0.005; n=1000.

**Table 3:** Reliability analysis of the screening tool.

Factors	Cronbach’s alpha	ICC	95% CI	*p*
Language expression	0.87	0.81	0.66-0.92	<0.0001
Language reception	0.91	0.91	0.88-0.93	<0.0001
Total	0.87	0.83	0.68-0.93	<0.0001

ICC, intraclass correlation coefficient; CI, confidence interval.

**Table 4:** Contingency table (N=962).

	Assessment (gold standard criteria)		
Screening	Failed	Passed	Total
Failed	99	9	108
Passed	21	833	854
Total	120	842	962

**Table 5:** Screening test: sensitivity, specificity, positive predictive value, negative predictive value, accuracy, and prevalence.

N	962
Sensitivity (%)	82.50
Specificity (%)	98.93
Positive predictive value (%)	91.3149
Negative predictive value (%)	97.6446
Accuracy	96.88
True disease prevalence (%)	12.47
Estimated disease prevalence: screening (%)	11.22

**Table 6:** Sociodemographic characterization of children who failed the language assessment (gold standard criteria) (n=120).

Demographic information	Classification	%
Age range	0-3 months	4.16
	4-6 months	3.33
	7-12 months	3.33
	1-2 years	19.16
	2-3 years	15
	3-4 years	20
	4-5 years	35
Sex	Male	69
	Female	31
Socioeconomic status*	A1	2
	A2	4.5
	B1	19.5
	B2	19.5
	C1	22
	C2	27
	D	3.5
	E	2

* Socioeconomic status according to Associa��o Brasileira de Empresas de Pesquisa (ABEP)-2009.

## APPENDIX

**Table:**

Threshold (to fail the screening)	Hearing and understanding (Language reception)	Talking (Language expression)
**Birth–3 Months** Two or more negative answers in language reception or language expression	• Startles at loud sounds. • Becomes quiet or smiles when you talk. • Seems to recognize your voice. Becomes quiet if crying.	• Makes cooing sounds. • Cries change for different needs. • Smiles at people.
**4–6 Months** Three or more negatives answer in language reception or language expression	• Moves eyes in the direction of sounds. • Responds to changes in the tone of your voice. • Notices toys that make sounds. • Pays attention to music.	• Coos and babbles when playing alone or with you. • Makes speech-like babbling sounds, like *pa*, *ba*, and *mi*. • Giggles and laughs. • Makes sounds when happy or upset.
**7 Months–1 Year** Four or more negatives answers in language reception or language expression	• Turns and looks in the direction of sounds. • Looks when you point. • Turns when you call her name. • Understands words for common items and people—words like *cup*, *truck*, *juice*, and *daddy*. • Starts to respond to simple words and phrases, like “No,” “Come here,” and “Want more?” • Plays games with you, like peek-a-boo and pat-a-cake. • Listens to songs and stories for a short time.	• Babbles long strings of sounds, like *mimi upup babababa*. • Uses sounds and gestures to get and keep attention. • Points to objects and shows them to others. • Uses gestures like waving bye, reaching for “up,” and shaking their head no. • Imitates different speech sounds. • Says 1 or 2 words, like *hi*, *dog*, *dada*, *mama*, or *uh-oh*. This will happen around the first birthday, but sounds may not be clear.
**1–2 Years** Three or more negative answers in language reception or language expression	• Points to a few body parts when asked. • Follows one-part directions, like “Roll the ball” or “Kiss the baby.” • Responds to simple questions, like “Who's that?” or “Where's your shoe?” • Listens to simple stories, songs, and rhymes. • Points to pictures in a book when you name them.	• Uses a lot of new words. • Uses *p*, *b*, *m*, *n, t, d, k, g, f,* and *v* in words. • Starts to name pictures in books. • Asks questions, like “What's that?”, “Who's that?”, and “Where's kitty?” • Puts two words together, like “more apple,” “no bed,” and “mommy book.”
**2–3 Years** Two or more negative answers in language receptionorFive or more negative answers in language expression	• Understands opposites, like go–stop, big–little, and up–down. • Follows two-part directions, like “Get the spoon and put it on the table.” • Understands new words quickly.	• Has a word for almost everything. • Talks about things that are not in the room. • Uses *s*, *z*, *ch*, *j*, *l*, and *nh* in words. • Uses words like *in*, *on*, and *under*. • Uses two or three words to talk about and ask for things. • People who know your child can understand him/her. • Asks “Why?” • Puts three words together to talk about things. May repeat some words and sounds.
**3–4 Years** Three or more negative answers in language receptionorFive or more negatives answers in language expression	• Responds when you call from another room. • Understands words for some colors, like *red*, *blue*, and *green*. • Understands words for some shapes, like *circle* and *square*. • Understands words for family, like *brother*, *grandmother*, and *aunt*.	• Answers simple who, what, and where questions. • Uses *lh, r, and rr* • Uses pronouns, like *I*, *you*, *me*, *we*, and *they*. • Uses some plural words, like *toys*, *birds*, and *buses*. • Most people understand what your child says. • Asks when and how questions. • Puts four words together. May make some mistakes, like “I goed to school.” • Talks about what happened during the day. Uses about four sentences at a time
**4–5 Years** Three or more negative answers in language receptionorFive or more negative answers in language expression	• Understands words for order, like *first*, *next*, and *last*. • Understands words for time, like *yesterday*, *today*, and *tomorrow*. • Follows longer directions, like “Put your pajamas on, brush your teeth, and then pick out a book.” • Follows classroom directions, like “Draw a circle on your paper around something you eat.” • Hears and understands most of what is heard at home and in school	• Says all speech sounds in words. May make mistakes on sounds that are harder to say, like *l* and *r* in consonant clusters and *r* and *s in final word position.* • Responds to “What did you say?” • Talks without repeating sounds or words most of the time. • Names letters and numbers. • Uses sentences that have more than one action word, like *jump*, *play*, and *get*. May make some mistakes, like “Zach gots 2 video games, but I got one.” • Tells a short story. • Keeps a conversation going. • Talks in different ways, depending on the listener and place. Your child may use short sentences with younger children. He may talk louder outside than inside.
